# Recyclability of Opaque PET from High Speed Melt Spinning: Determination of the Structures and Properties of Filaments

**DOI:** 10.3390/polym14112235

**Published:** 2022-05-31

**Authors:** Félix Odet, Noëllie Ylla, René Fulchiron, Philippe Cassagnau

**Affiliations:** Univ-Lyon, Université Claude Bernard Lyon 1, Ingénierie des Matériaux Polymères, CNRS, UMR 5223, 15 Bd Latarjet, 69622 Villeurbanne, France; felix.odet@freudenberg-pm.com (F.O.); noellie.ylla@univ-lyon1.fr (N.Y.); rene.fulchiron@univ-lyon1.fr (R.F.)

**Keywords:** polyethylene terephthalate (PET), opaque rPET, recycling, TiO_2_, crystallinity, tenacity, mesophase, high-speed spinning, filament morphology, molecular orientation

## Abstract

Recycling opaque Polyethylene terephthalate (PET), which contains 1 to 10 wt % TiO_2_ submicron particles, has become of interest in the past few years. However, the bottle-to-fiber recyclability of opaque PET has not been assessed yet. In this work, opaque PET packaging has been characterized, and high-speed melt-spun filaments with different amounts of opaque PET (30–50–100%) blended with standard transparent recycled PET (rPET) have been produced in a pilot system. The opaque PET filaments produced have also been compared to a transparent rPET blend with masterbatch PET/TiO_2_ at different amounts of filler (1–3–6 wt %), produced with the same parameters. The structure-properties relationship of rPET melt-spun fibers has been investigated with crystallinity measurements, amorphous and crystalline phases orientation, and tenacity. It has been observed that the degree of crystallinity, the crystalline and amorphous phases orientation and the tenacity decreases with opaque PET addition and, to a lesser extent, with TiO_2_ addition. It has been suggested that TiO_2_ particles are not entirely responsible for the decrease in mechanical properties of opaque PET filaments since opaque rPET filaments have inferior properties to r-PET/TiO_2_ filaments at the same filler content.

## 1. Introduction

When considering the environmental issues of the current period in terms of pollution, extraction of non-renewable resources, and greenhouse gas emission, recycling packaging becomes more and more necessary. In this context, improving the recycling rate of packaging is a topic of interest; with rates at 42% and 24% in 2019 in Europe and the U.S, respectively, 58% and 29% for PET more specifically [1,2]. Fiber production is the most important application for recycled polyethylene terephthalate (r-PET), representing 40% of the market volume in 2016 [3]. Since packaging formulation is in constant evolution, recyclers need to adapt their process continuously. Recently, opaque PET bottles have emerged in the European market, mainly for milk bottles. Formerly in HDPE, the new technology using PET is filled with submicron ${\mathrm{TiO}}_{2}$ particles, and to a lesser extent, carbon black. It is lighter, does not need aluminum sealing, and is faster to produce. Thus, its environmental impact is reduced in terms of energy and water consumption, from 30% and 20%, respectively. However, the presence of filler may disturb the recycling activity, especially in the spunbond industry, producing filaments of a few micrometers in diameter. The impact of this new packaging on the melt spinning process and the fiber properties need to be evaluated.

In the high-speed melt spinning process, filaments going out of a capillary die undergo very high extensional strain rates. It generates molecular orientation and stress-induced crystallization responsible for the final mechanical properties, mainly tenacity and dimensional stability [4,5,6]. The process stability requires a precise range of molar mass and rheological behavior [6]. In addition, the PET filaments’ properties are mainly driven by process parameters, such as strain rate, i.e., spinning speed, as studied by many authors [6,7,8,9].To a lesser extent, it also depends on the raw material properties, such as molar mass [6,7,8,9] or the presence of filler [10,11,12,13,14,15]. A higher molar mass implies higher stress in the spinline and higher relaxation time, resulting in higher molecular orientation and crystallinity [6,8]. The influence of nano-particles in PET melt-spun fiber is diversified, depending on the study conditions and filler content. Some authors observe the reduction of mechanical properties due to poor orientation and crystallization [14,16,17], and some note better properties at low concentrations of nano-filler [15,18,19]. More specifically, the influence of ${\mathrm{TiO}}_{2}$ on high-speed spinning has rarely been studied. However, some authors [10] observe an anti-nucleating effect of ${\mathrm{TiO}}_{2}$ on stress-induced PET crystallization, whereas ${\mathrm{TiO}}_{2}$ has a nucleating effect on non-isothermal slow crystallization. Finally, recycled opaque PET (O-rPET) influence on high-speed spinning has not been reported yet. This work attempts to evaluate mechanical recyclability via the high-speed spinning process of commercial opaque PET bottles and understand the origin of the O-rPET filaments’ structure and properties.

The impact of O-rPET on melt-spun fiber structure and properties will be assessed and compared to the influence of ${\mathrm{TiO}}_{2}$ incorporated via a PET/${\mathrm{TiO}}_{2}$ masterbatch. The aim is to discriminate the contribution of the particles and the opaque PET matrix to the filament properties. Firstly, raw O-rPET and classical rPET material were characterized according to their molar mass, filler content and nature, and rheological behavior in elongation. Then, melt-spun filaments of rPET, rPET with ${\mathrm{TiO}}_{2}$ from the masterbatch, and O-rPET were produced in an industrial pilot system. Filaments were characterized according to their structure and mechanical properties.

## 2. Materials and Methods

### 2.1. Materials

#### 2.1.1. Raw Materials

rPET transparent flakes, named “standard rPET” in this study, came from Freudenberg Performance Material (Colmar, France) and were used as the reference. This rPET grade is constituted by various post-consumer PET bottles, mainly colored, some opaque and transparent. The post-consumer bottles undergo selective sorting, washing, drying, and grinding, allowing the flakes to contain less than 100 ppm PVC, 25 ppm metals, 20 ppm textile, and 150 ppm polyolefin.

Opaque rPET flakes from milk bottles were used, coming directly from the injection blow-molding process of industrial collaborators of Freudenberg Performance materials (Colmar, France) without being used by consumers. It contained 5.7 wt % of ${\mathrm{TiO}}_{2}$. The different properties of raw materials are shown in Table 1.

$\eta_{0}\;$As described in Table 1, the intrinsic viscosity of opaque PET was lower than the reference one. On the other hand, crystallization temperatures of raw materials were slightly different, with higher temperatures for opaque PET compared to the reference. It suggests a nucleating effect of ${\mathrm{TiO}}_{2}$ on crystallization, as observed by various authors [10].

#### 2.1.2. Extruded Blends

For high-speed melt spinning, different blends of standard rPET and opaque rPET were produced by extrusion, as summarized in Table 2. In another part, standard rPET with several amounts of ${\mathrm{TiO}}_{2}$ were extruded, as detailed in Table 3. The ${\mathrm{TiO}}_{2}$ used in these blends was a PET/${\mathrm{TiO}}_{2}$ 50/50 masterbatch Cromomix 80,068 provided by ICAP Masterbatch.

TGA analysis was conducted to determine the filler content of each extruded blend shown in Table 2 and Table 3. The REF, composed of standard rPET, contained about 0.8% filler. The 100%-opaque sample contained about 5.7%, mostly ${\mathrm{TiO}}_{2}$. The other blends had intermediate filler contents. The formulations with ${\mathrm{TiO}}_{2}$ from the masterbatch had a similar filler content as the previous formulations, from 1.8 wt % to 6.8 wt %.

### 2.2. Processing Methods

At first, PET samples were dried before any melt processing and analysis in the melted state. The drying was performed in a vacuum oven at 115 °C for 15 h.

#### 2.2.1. Extrusion

Blends, defined in Table 2 and Table 3, were produced via the extrusion process. A twin-screw extruder (Leistritz, Nuremberg, Germany; Diameter D = 18 mm with an L/D of 60) was used at 275 °C, with a flow rate of 4 kg/h and a screw rotational speed of 300 rpm. Before blending, each sample, i.e., standard rPET and O-rPET mix, was extruded separately to transform the flakes into pellets at 275 °C (operating conditions: 4 kg/h and 800 rpm).

#### 2.2.2. Melt Spinning

High-speed melt spinning was carried out in a pilot system from Freudenberg Performance Materials R&D (Weinheim, Germany) with a spin pack of 4 dies of 0.4 mm diameter. Samples were melted at 285 °C in a transport screw before passing through a gear pump, defining the throughput of material coming into the die. The throughput (Q) was fixed at 2.5 g/hole/min, corresponding to $2.1\;{\mathrm{cm}}^{3}/\min$, since the melt PET density was 1.17 [16]. The filaments underwent drawing to an aerodynamic injector with a pressure established at 5.4 bar. It corresponded to a take-up speed of 6000 m/min, producing filaments of 4.6 dtex. The following equations describe the high-speed spinning test:

- Initial velocity $V_{0}$ of filament:
  (1) $$V_{0}=\frac{Q}{\pi R_{0}^{2}}=\frac{{2.1\times 10}^{-6}}{\pi \times {\left({2\times 10}^{-4}\right)}^{2}}$$

With the initial radius of the filament $R_{0}={2\times 10}^{-4}\;m$.

- Hencky strain $\;\varepsilon_{H}$:
  (2) $$\varepsilon_{H}=\ln\left(\frac{R_{0}^{2}}{R_{f}^{2}}\right)=5.9$$

With $R_{f}$ the radius of the drawn filament, measured at 10.5 µm and calculated with the titer (4.6 dtex) and the density of the filament (1.37) according to Equation (3):

(3) $$R_{f}=\sqrt{\frac{titer}{density}\times \frac{1}{\pi }}=\sqrt{\frac{4.6}{1.37}\times \frac{1}{\pi }}=10.35\;µm$$

- Draw ratio (DR):
  (4) $$DR=\frac{V_{f}}{V_{0}}=e^{\varepsilon_{H}}=360$$

- Take-up speed $V_{f}$:
  (5) $$V_{f}=DR\cdot V_{0}\approx 6000\;m/\min$$

### 2.3. Characterization Methods

#### 2.3.1. TGA

The filler content of each material was determined by mass loss measurement on a TGA Q500 (TA instrument, Waters technologies Corporation, Massachusetts, MA, USA). A 10 °C/min ramp was applied from ambient to 660 °C, with an airflow of 40 mL/min. The mass loss between 200 °C (to deal with dried sample) and 660 °C represented the matrix content. The remaining mass corresponded to the inorganic filler content.

#### 2.3.2. SEM/EDX

The morphology of the raw materials filler was examined using a scanning electron microscope (SEM, Zeiss, Oberkochen, Germany) model Zeiss compact at 10 keV with a secondary electron detector. The samples, TGA residual ashes of the raw materials, were coated with copper before the analysis to reduce charging effects. SEM-EDX spectroscopy analyses were carried out using the Zeiss compact microscope with the same conditions to determine the crystalline nature of the filler, using an SDD detector Oxford of 50 mm².

#### 2.3.3. DSC

A DSC Q200 (TA Instrument, Waters technologies Corporation, Massachusetts, MA, USA) was used to measure the melting and crystallization temperatures of samples. Two cycles were run, composed of a heating ramp from ambient to 275 °C at 10 °C/min, followed by a cooling ramp from 275 °C to ambient.

The crystallinity of each filament produced by melt spinning was determined with a DSC Q200. A 10 °C/min ramp was applied from ambient to 275 °C with a nitrogen flow of 40 mL/min. The degree of crystallinity of melt-spun filaments was measured according to the equation:

(6) $$X_{c}\left(\%\right)=100\times \frac{\;\Delta H_{m}-\Delta H_{cc}}{\Delta H_{m}^{th}}\;$$

With the enthalpy of fusion $\Delta H_{m}=\frac{\Delta H_{m}^{measured}}{1-\varphi }$ and the enthalpy of cold crystallization $\Delta H_{cc}=\frac{\Delta H_{cc}^{measured}}{1-\varphi }$.

Where φ is the mass concentration of the filler. For PET, the theoretical enthalpy of fusion $\Delta H_{m}^{th}$ for a fully crystalline PET is 120 J/g [10].

#### 2.3.4. Rheology/Absolute Complex Viscosity

A DHR rheometer from TA Instruments (Waters technologies Corporation, Massachusetts, USA) was used with a 25 mm diameter plate/plate geometry under slight airflow (10 mL/min). The absolute shear complex viscosity ($\left|\eta^{*}\left(\omega \right)|\right)$ was determined with a frequencies sweep analysis. Frequencies sweeps were carried out at 280 °C, 10% deformation, 100 rad/s to 0.1 rad/s. The analysis was done from high frequencies to low frequencies to measure several points in the first minutes of the experiment, where no degradation can occur. Since the points measured at low frequencies are consistent with those at high frequencies, it can be assumed that no degradation occurs during the experiment. Mean molar masses were assessed from the inverse rheological method using the frequencies sweep analysis developed by Tuminello et al. [17]. Three measurements were made for the samples of the raw materials. The average curve is used in the result section.

#### 2.3.5. Intrinsic Viscosity

Intrinsic viscosity (IV) was measured with an Ubbelohde viscometer (Merck KGaA, Darmstadt, Germany) at 25 °C. Samples were solubilized in phenol/dichlorobenzene at 125 °C for 20 min with a polymer concentration of 5 g/L. The filler weight fraction of each specimen was deducted to maintain the polymer concentration at 5 g/L. IV was calculated from Ciuta et al.’s equation [18]:

(7) $$IV\;\left(\frac{dL}{g}\right)=\underset{C\to 0}{\lim}\left(\frac{\frac{t-t_{0}}{t_{0}}}{C}\right)=\frac{{\left(2\times \left(\frac{t-t_{0}}{t_{0}}-\ln\left(\frac{t}{t_{0}}\right)\right)\right)}^{\frac{1}{2}}}{C}$$

where $t_{0}$ is the elution time of the solvent, t is the evolution time of the solution containing the dissolved sample, and *C* is the concentration of the sample in the solvent. Once solubilized, the samples were filtered with a 0.4 µm mesh size filter to prevent the capillary from being obstructed by the filler. The transparency of the solution obtained compared to its initial opacity indicated a strong reduction of the filler concentration.

#### 2.3.6. Melt Strength

The elongational rheology behavior of PET was complex to study using classical methods due to sample sagging [19,20,21], and there were difficulties in describing the spinning process, as it was strongly non-isothermal with high and inconstant strain rates along the spinning line [20,22]. Another approach was to use a haul-off system in a capillary rheometer for qualitative measurement from Malvern instruments (Malvern, Great Britain). The filament coming out of the capillary at a speed $v_{0}\;$passes through two pulleys and is drawn with a wheel at a controlled speed $v_{f}$. The tension of the filament is measured on the first pulley using a weighing scale. Hence, the tensile draw-down force can be measured versus the take-up speed, frequently presented as the melt strength and the draw ratio. This experiment is closer to the melt spinning industrial process than usual methods. A qualitative spinnability can be observed with the tensile drawn-down force and the maximal draw ratio before breaking (DR) [22,23].

The experiment was carried out at 275 °C, with a die of 0.5 mm in diameter and 10 mm in length. The piston speed was fixed at 0.083 mm/s, causing a shear rate of 1100/s at the wall of the capillary die and a filament initial velocity${\;v}_{0}=0.075\;m/s$. The haul-off wheel speed was increased at 0$.025\;m/s^{2}$ until the breakage of the filament. Moreover, three measurements were made for the samples of the raw materials. The average curve was used in the result section.

#### 2.3.7. WAXS

The filaments’ crystalline and amorphous orientations were characterized using Wide Angle X-ray Scattering (WAXS) analysis. These experiments were carried out in a transmission mode on a Gemini diffractometer (Centre de diffractométrie Longchambon, UCBL, Villeurbanne, France). The voltage of the X-Ray copper tube was 50 kV, and the wavelength of the beam-line was 1.54 Å. The WAXS patterns were recorded by scanning 2θ from 5° to 50° with a CDD ATLAD detector of 135 mm diagonal size and 48 µm per pixel.

The Hermans orientation factor for the c-axis of the crystalline cell, $f_{c}$, was determined according to the Wilchinsky approach applied to PET by Gupta et al. [24]. It requires the azimuthal scan of the three crystalline planes (010), ($1\bar{1}0$), and (100), all containing the c-axis. The intensity diffracted by these planes was recorded as a function of the azimuthal angle Φ, by averaging between 2θ = 16.7 to 18.7, 2θ = 21.5 to 24 and 2θ = 24.1 to 26.6 for the three planes, respectively. Then, $f_{c}\;$was determined using the following formula:

(8) $$f_{c}=1-\frac{3}{2}\sin^{2}\chi$$

where χ is the angle between the c axis of the unit cell and the fiber axis. sin²χ was calculated using the formula from Gupta et al. [24]:

(9) $$\sin^{2}\chi =0.356\cos^{2}\Phi_{010}+0.767\cos^{2}\Phi_{1\bar{1}0\;}+0.877\cos^{2}\Phi_{100}$$

where cos²Φ is determined for each of the three considered planes, according to the following equation [24]:

(10) $$cos²\Phi =\frac{\int_{0}^{\frac{\pi }{2}}I\left(\Phi \right)cos^{2}\Phi \;sin\Phi \;d\Phi }{\int_{0}^{\frac{\pi }{2}}I\left(\Phi \right)\;sin\Phi \;d\Phi }$$

where I(Φ) is the diffracted intensity as a function of the azimuthal angle Φ with Φ=0 corresponding to the fiber axis.

$f_{c}=1\;$corresponds to a perfectly oriented c-axis of the crystalline phase along the fiber axis, whereas $f_{c}=0\;$corresponds to a totally disoriented phase and $f_{c}=-0.5$ to a perfectly oriented c-axis perpendicular to the fiber axis.

Transmission WAXS analysis was also used to characterize the highly oriented amorphous phase orientation, according to Wu et al.’s methods [25]. The literature generally describes filament morphology as three distinct phases. In addition to the crystalline and amorphous phase, there is a highly oriented amorphous phase, called mesophase, taut tie molecules, intermediate phase, or oriented non-crystalline phase [25,26,27,28,29]. This phase, consisting of extended molecular chains along the fiber axis, is of primary importance since it drives the mechanical properties of the fiber [30]. The orientation of the chain creates anisotropy, which can be observed in WAXS measurement with the variation of the intensity of the amorphous halo along the azimuthal angle.

Wu et al.’s method involved measuring the anisotropy of the amorphous x-ray halo along the azimuthal angle, giving the amount and the orientation of the mesophase. The intensity versus *2θ* from 5° to 35° was plotted for several azimuthal angles, from 0 (along the fiber axis) to 90° every 10°. For each scan, the crystalline contribution was subtracted by spectral deconvolution with Fityk software ( version 0.9.8, General Public License [31]). The area of the amorphous intensity signal A(Φ) was then recorded for each scan, normalized to the area of the amorphous intensity signal at the 0° scan representing the isotropic amorphous phase $A_{iso}\left(\Phi \right)$, and plotted against the azimuthal angle. This plot depicted the evolution of the amorphous signal intensity versus the azimuthal angle. It gave the amount and orientation of the mesophase since the isotropic amorphous phase contribution was constant over the azimuthal angle. The fraction of the mesophase $X_{meso}$ among the amorphous phase can be determined with the integration of this plot with [25]:

(11) $$X_{meso}=100\times \int_{\Phi =0}^{\Phi =90}\left(\frac{\left(\;A\left(\Phi \right)-A_{iso}\left(\Phi \right)\right)}{A\left(\Phi \right)}\;\right)d\Phi$$

The molecular orientation of the non-crystalline phase was usually determined using birefringence measurement or Raman spectroscopy analysis, as explained elsewhere [10,29,32,33]. Both methods were attempted in this work but will not be presented. Indeed, ${\mathrm{TiO}}_{2}$ presence tends to opacify the samples so that light cannot pass through the filament, making the birefringence analysis impossible. In addition, the opaque PET samples used in this study contain carbon black, which generates significant degradations of the matrix during the Raman spectroscopy analysis, leading to a poor signal-to-noise ratio.

#### 2.3.8. Stress-Strain Curves and Tenacity

The tensile properties and the titer of the filaments were determined using a Lenzing AG dynamometer (Lenzing, Austria). The gauge length and crosshead speed were 20 mm and 50 mm/min, respectively. The samples were prepared in the form of single filaments. Ten filaments were analyzed; an average stress-strain curve was obtained from each sample.

## 3. Results and Discussion

### 3.1. Raw Materials and Extruded Samples

**Figure 1:**
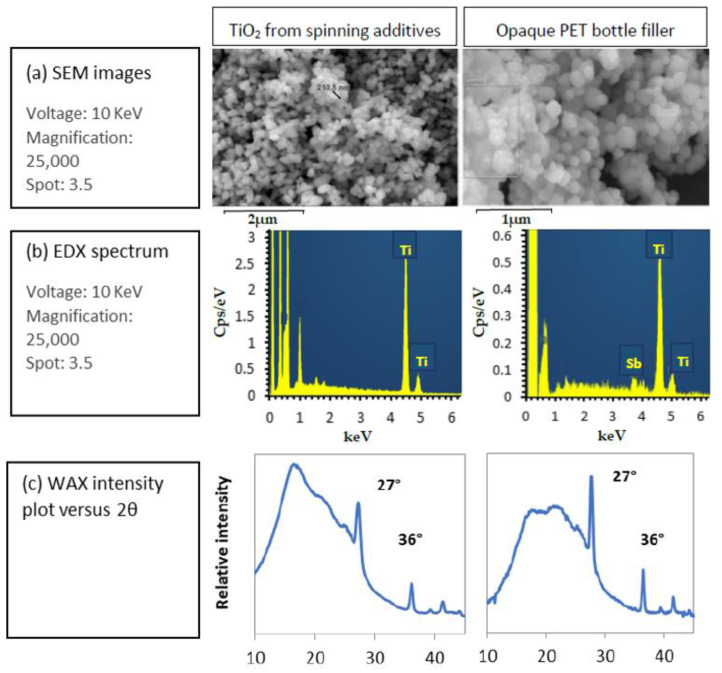
Characterization of TiO_2_ particles from fiber industry additives (**left**) and bottle industry additives (**right**) with (**a**) SEM images, (**b**) EDX spectrum and (**c**) WAX intensity plot.

Opaque PET filler was analyzed using SEM and compared to the PET/${\mathrm{TiO}}_{2}$ 50/50 masterbatch used in the textile industry. Opaque PET contains mostly ${\mathrm{TiO}}_{2}\;$spherical particles of 200 nm diameter according to SEM images of TGA ashes and their corresponding EDX spectra (Figure 1a,b, respectively). Indeed, a huge peak at 4.5 keV is present for both samples, corresponding to the energy of the Kα X-rays of Titanium. Antimony is also detected in the spectra at 3.7 keV at a low concentration for opaque rPET and standard rPET bottles since it is a PET polyaddition catalyst. Kim et al. [34] observed that Antimony-based catalysts are more convenient than others for spinning applications. In addition, wide-angle X-Ray analysis of both samples presented in Figure 1c shows crystalline phases at 2θ=27 and 36° corresponding to ${\mathrm{TiO}}_{2}\;$rutile form [35].${\;\mathrm{TiO}}_{2}$ from the textile industry masterbatch shows the same characteristic of rutile particles of 200 nm diameter. Therefore, the nature of the filler in opaque PET bottles is similar to that of ${\mathrm{TiO}}_{2}$/PET masterbatch used in the textile industry as a delustrant [27,36].

**Figure 2:**
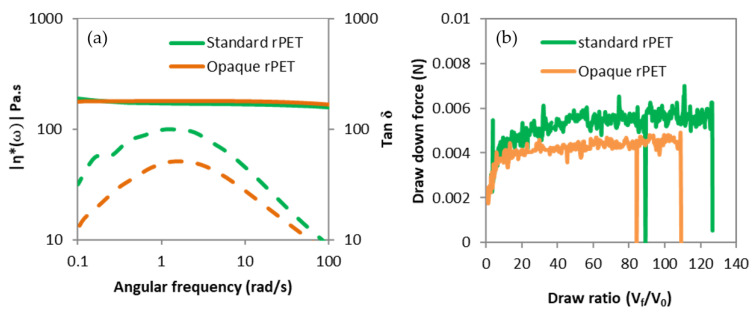
Rheological characterization of standard rPET and opaque rPET raw samples, with (**a**) Absolute complex viscosity and tan δ (dotted line) versus frequency, and (**b**) Draw-down force versus draw ratio.

On the other hand, the raw materials have been characterized according to their shear and elongational rheological behavior, as shown in Figure 2. The zero-shear viscosity of raw materials is shown in Table 1. Both samples show a Newtonian behavior and an equivalent complex viscosity, whereas the intrinsic viscosity of opaque rPET is lower (see Table 1). It is due to matrix-particle interactions which increase the low-shear viscosity [37]. In addition, Tan δ is lower for opaque rPET for an equivalent viscosity and a lower molar mass than standard rPET. It means there is higher elastic behavior [38], which can provoke poorer spinning behavior [39]. Elongational rheology has been characterized via haul-off analysis, as described in Section 2.3. The draw ratio (DR), defined as $V_{f}/V_{0}$, and the melt strength, defined as the draw-down force at break, shown in Figure 2b, are commonly used to characterize the melt spinnability of a polymer. The DR describes the drawability of the polymer melt, which is of primary importance in melt spinning since the DR is settled at a high value, around 400. Qualitatively, the higher the melt strength, the higher the stress in the filaments at fixed DR during drawing. The stress in the spinline drives molecular orientation and crystallization. Lower stresses can induce a decrease in molecular orientation and, consequently, poor filament properties. On the other hand, higher stresses can lead to the breakage of the filament [24,26,27].

It can be observed that opaque PET raw material has a lower draw-down force and lower draw ratio before breaking than the standard rPET. Opaque PET spinnability looks poorer than standard rPET, probably due to the lower molar mass, ${\mathrm{TiO}}_{2}$ presence, or both. However, industrial and pilot spinlines induce draw ratio and elongational strain rates far higher than the haul-off system. Consequently, the behavior observed in the haul-off system cannot be simply generalized to spinning systems.

Furthermore, the different extruded systems’ intrinsic viscosity and the rheological behavior in shear have been characterized. Intrinsic viscosity presented in Table 2 and Table 3 is very similar between samples, around 0.55 dL/g, but is lower in raw samples (0.82 and 0.73 for standard and opaque PET, respectively). Degradations occur during extrusion, leading to a similar molar mass for each blend. Thus, opaque rPET and ${\mathrm{TiO}}_{2}$ have no negative influences on matrix stability during extrusion. One can observe a slight decrease between the REF and the filled samples with ${\mathrm{TiO}}_{2}\;$from the masterbatch. It is probably due to the polymeric matrix of the masterbatch, which has a lower molar mass than the reference. 

**Figure 3:**
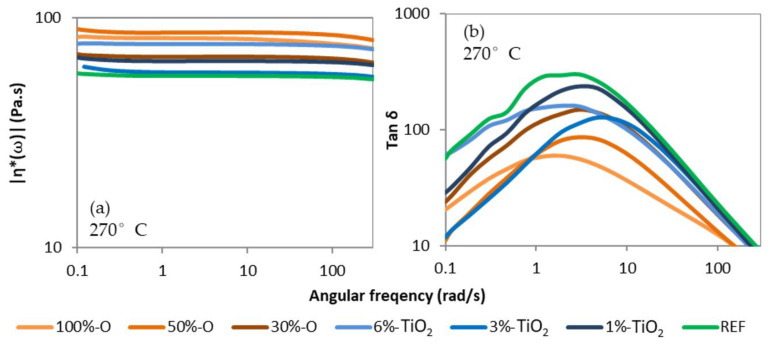
(**a**) Absolute complex shear viscosity, and (**b**) tan δ, of extruded blends. Measurements realized at 270 °C.

The shear viscosity at 270 °C at low shear frequencies shown in Figure 3a is lower than in raw materials due to the thermo-oxidative degradations during extrusion. The viscosity is similar between each formulation, from 55 to 85 Pa.s in the Newtonian plateau. A slight viscosity increase with the filler concentration can be observed, due to particle-matrix interactions, as described in the literature [37]. Thus, the REF has the lowest shear viscosity but has an equivalent intrinsic viscosity to the other systems due to the absence of filler. However, these interactions are not critical since the viscosity remains in the same order of magnitude. When looking at Figure 3b, the opaque blends have a lower tan δ, i.e., higher elastic behavior than the REF and standard rPET systems filled with ${\mathrm{TiO}}_{2}$. Thus, the opaque rPET matrix has a higher elastic behavior than the standard rPET, independent of the filler. Some authors observed that elastic behavior could be detrimental to melt spinning application, reducing the stability of the process. Thus, tan δ should be as low as possible [39].

Finally, due to degradation during extrusion, the elongation rheology of the extruded blends could not be studied with haul-off experiments that require sufficient viscosity.

### 3.2. PET Melt Spinning Filaments

Spinning tests were performed with the extruded blends described in Table 2 and Table 3, constituting two sets of systems. The objective was to investigate and compare the influence of both opaque PET and ${\mathrm{TiO}}_{2}$ from textile grade masterbatch additions in standard rPET. The filaments of these two sets of systems were characterized according to their molecular orientation, degree of crystallinity, and tenacity.

#### 3.2.1. Crystallinity

**Figure 4:**
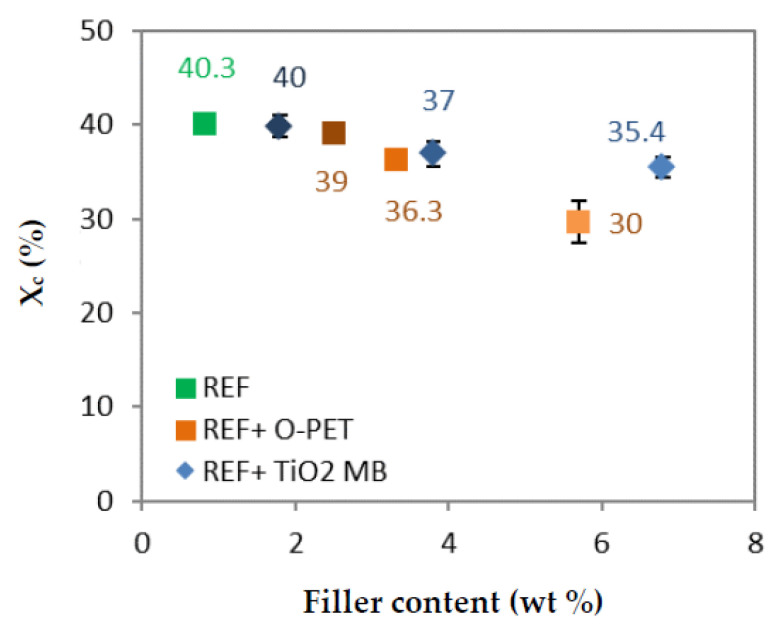
Degree of crystallinity (%) versus the filler content (wt %) for the two sets of filaments and the reference (green).

Table 4 shows the degree of crystallinity of each filament. The reference has the highest degree of crystallinity, at 40%, while the other blends have a lower degree of crystallinity, until 30% for the 100%-opaque sample. Figure 4 shows the degree of crystallinity versus the filler concentration for the two sets of systems. It can be seen that the addition of both ${\mathrm{TiO}}_{2}$ and opaque PET in standard rPET results in a decrease in the degree of crystallinity proportionate to the filler content. However, opaque PET has a more negative influence on crystallization at an equivalent filler content. Its reduced crystallinity is due to a lower stress-induced crystallization phenomenon during spinning, according to the literature [5,7,28]. The lower stress during spinning can be caused by the sub-micron ${\mathrm{TiO}}_{2}$ physical presence, as observed by Taniguchi et al. [10]. In addition, lower stress in the spinline is also caused by a lower elongational viscosity [27,40,41]. As Figure 2 suggests that the elongational viscosity of opaque PET is lower than the reference, this may account for the lower crystallinity of opaque PET compared to the blends of rPET filled with ${\mathrm{TiO}}_{2}$ from masterbatch. As a result, opaque PET may show a lower degree of crystallinity because of its filler content and lower elongation viscosity.

One can observe that no correlation can be found between the degree of crystallinity of raw materials and extruded blends and those with the corresponding filaments. The nucleating effect of ${\mathrm{TiO}}_{2}$ in non-isothermal crystallization is not observed in the stress-induced crystallization present in the melt spinning process, as observed by several authors [10,14].

#### 3.2.2. Molecular Orientation

**Figure 5:**
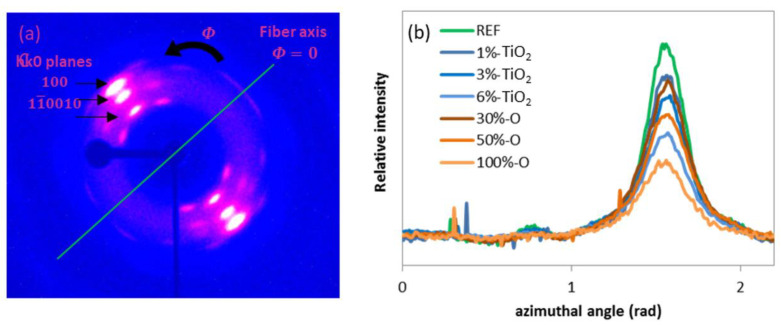
(**a**) WAXS 2D pattern of the reference and (**b**) X-ray intensity of crystalline plane 100 versus the azimuth angle.

**Figure 6:**
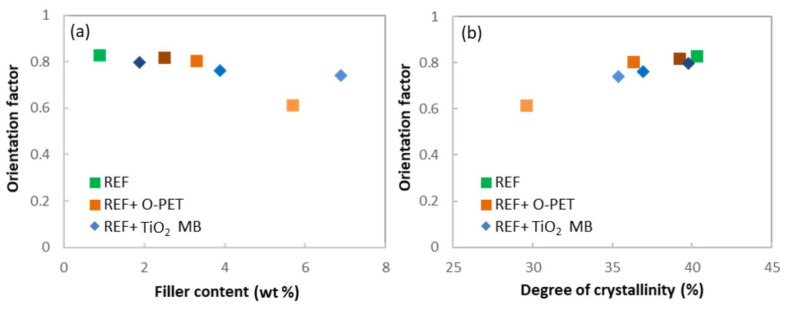
(**a**) Orientation factor (f_c_) versus filler content (wt %) and (**b**) Orientation factor versus degree of crystallinity (%); for the two sets of filaments and the reference (green).

The orientation of the crystalline structure was characterized via transmission WAXS analysis, whose reference 2D pattern is presented in Figure 5a. The intensity of the X-Ray scattering versus the azimuthal angle is shown in Figure 5b for plane 100 (*2θ* = 26.5°). The Hermans’ orientation factor (fc) is exposed in Table 4, calculated with the method developed by Gupta et al. [24] and exposed in Section 2.3. The orientation factor versus the filler content is plotted in Figure 6a.

It can be seen that the relative maximum intensity of the X-ray peak of the hk0 plane (Figure 5b), and consequently the orientation factor (Figure 6a), decreases with opaque PET and ${\mathrm{TiO}}_{2}$ addition. The 100% opaque sample orientation factor is particularly weak compared to the other blends. The orientation factor decreases more regularly with ${\mathrm{TiO}}_{2}$ concentration. In addition, the orientation factor is plotted versus the degree of crystallinity in Figure 6b. One can see a clear correlation between these characteristics: the orientation factor increases with the degree of crystallinity. This finding can be related to the mechanism of stress-induced crystallization occurring in melt spinning. Indeed, the molecular orientation initiates stress-induced crystallization beyond a certain take-up speed, around 4000 m/min [6,7,27]. Higher molecular orientation generates higher crystallinity with well-oriented crystallites. Samples with a high degree of crystallinity and crystallite orientation may imply high molecular orientation during spinning. On the contrary, samples with a weak degree of crystallinity and crystalline orientation imply a weaker molecular orientation, barely enough to start the crystallization with non-perfectly oriented crystallites. Thus, the degree of crystallinity and crystallite orientation are linked and controlled by molecular orientation, which is reduced by opaque PET and ${\mathrm{TiO}}_{2}$ addition. As for the degree of crystallinity, the opaque PET result is lower than the reference filled with ${\mathrm{TiO}}_{2}$. This finding confirms that ${\mathrm{TiO}}_{2}$ is not the only factor modifying the spinning process: the opaque PET matrix also has an impact.

**Figure 7:**
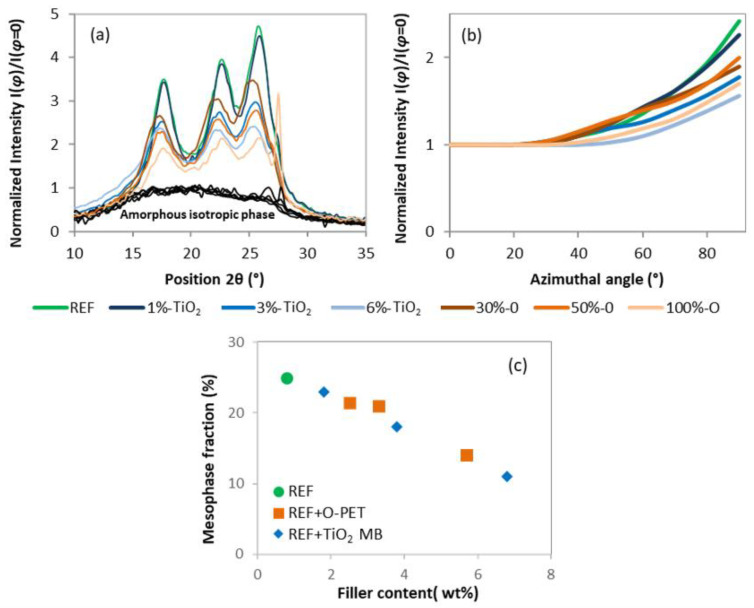
(**a**) Relative X-ray Intensity of the scans at Φ=90° from 10° to 35° normalized to the intensity of the scan at Φ=0° (black curves) corresponding to the amorphous isotropic phase; (**b**) Intensity of X-ray amorphous halo versus azimuthal angle, normalized to the intensity at Φ=0°; (**c**) Mesophase fraction (%) versus filler content (wt %).

The orientation and the quantification of the mesophase were also characterized via transmission WAXS analysis and presented in Figure 7, according to Wu et al.’s method described in Section 2.3 [25]. The intensity of the whole x-ray halo from 5° to 35° of the scans at Φ=90°, i.e., the maximum intensity direction, was normalized to the intensity of the scan at Φ=0°, i.e., the minimum intensity direction, and plotted in Figure 7a. The scan at Φ=0° is supposed to be totally non-crystalline and non-oriented as it is the amorphous isotropic phase contribution only. Figure 7a shows the differences between this amorphous isotropic phase (black) versus the maximal x-ray intensity coming from both the crystalline phase and mesophase. These differences give indications of the orientation of the crystalline phase and mesophase, which decreases with opaque PET and ${\mathrm{TiO}}_{2}$ addition in a similar way to the degree of crystallinity or crystalline orientation. In addition, the intensity of the amorphous halo only, which contains the oriented and non-oriented amorphous phases, versus the azimuthal angle was normalized to the intensity at Φ=0° and plotted in Figure 7b. The crystalline contribution was subtracted for each scan so that the increasing intensity is only due to the mesophase. As we approach the perpendicular of the fiber axis, the amorphous intensity increases, revealing the presence of the mesophase. The scan at 90° of the fiber axis presents the highest intensity; the mesophase contribution is maximal. It can be seen from Figure 7b that the reference has the highest intensity at 90° and the steepest curve, i.e., the highest orientation and amount of the mesophase. When opaque PET or ${\mathrm{TiO}}_{2}$ is added, the curves flatten, meaning a lower amount and a lower orientation of the mesophase. This decrease in molecular orientation can explain the one in both crystalline orientation and crystallinity and can induce lower tenacity, as described above. The mesophase fraction was more precisely calculated from the area of the curve in Figure 7b and plotted versus the amount of filler in each sample in Figure 7c. As already observed, the mesophase fraction is maximal with the reference and strongly decreases with PET opaque and ${\mathrm{TiO}}_{2}$ addition. However, unlike the evolution of crystallinity and crystalline orientation of opaque PET and standard rPET+${\;\mathrm{TiO}}_{2}$ samples that decrease at a different rhythm, all samples behave similarly according to their filler content. It could mean that the mesophase fraction depends only on the filler content and not on the matrix composition, with opaque PET behaving the same as standard rPET at the same filler content. However, the mesophase characterization involves the subtraction of the crystalline contribution to the X-ray pattern, which is complex to do accurately, especially in 40% crystalline samples. This analysis may not be as accurate as it needs to be to assert this hypothesis. When looking at Figure 7a, where no subtraction was performed, the differences between opaque rPET and standard rPET + ${\mathrm{TiO}}_{2}$ reappear.

#### 3.2.3. Mechanical Properties

**Figure 8:**
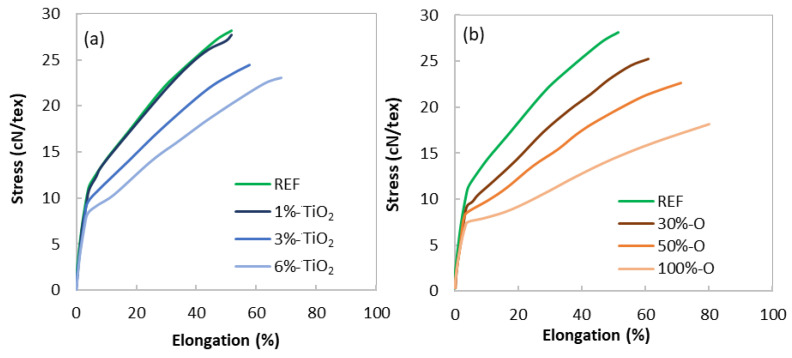
Stress versus elongation of filaments; (**a**) REF + TiO_2_ from masterbatch blends; (**b**) Standard rPET + opaque rPET blends.

**Figure 9:**
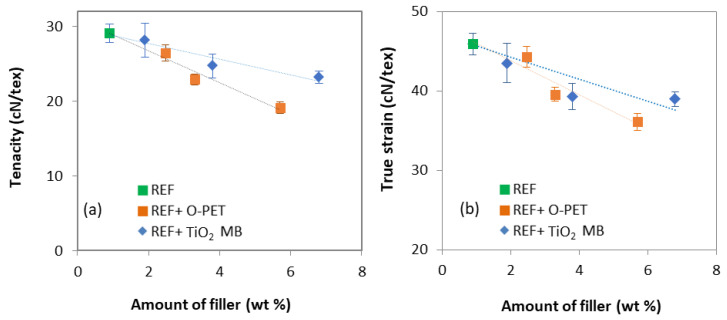
(**a**) Tenacity and (**b**) true strain versus the amount of filler for the two sets of filaments.

Stress versus elongation curves are shown in Figure 8, describing the influence of ${\mathrm{TiO}}_{2}$ (a) and the influence of opaque PET (b) addition in rPET. The value at the break, i.e., the tenacity, is reported in Table 4. Tenacity decreases when ${\mathrm{TiO}}_{2\;}$addition is more than 1 wt % and when opaque PET is added from 30%. It can be observed in Figure 9a, showing the tenacity versus de amount of filler, that opaque PET addition has a more negative influence on tenacity than ${\mathrm{TiO}}_{2}$ addition, for the same amount of filler in the matrix.

In addition, Figure 9b shows the product of tenacity and residual elongation at break called the true stress at break $\sigma_{b}$ versus the content of filler, calculated with the following equation [42]:

(12) $$\sigma_{b}=Tenacity\times \left(\frac{Elongation\left(\%\right)}{100}+1\right)$$

According to Beyreuther et al. [41], $\sigma_{b}\;$is more independent of process parameters than tenacity and is primarily related to the material properties. One can note that the trend is the same but less significant. Adapting the process parameter by increasing the draw ratio can improve the toughness of the opaque PET filaments, but without reaching those of the REF + ${\mathrm{TiO}}_{2}$ samples. However, spinning process stability does not allow an increased draw ratio for opaque PET samples. Thus, they require more stress in the spinline to get closer to REF +${\;\mathrm{TiO}}_{2}$ samples; meanwhile, they are less spinnable.

It is well known that filaments’ mechanical properties are mainly driven by the mesophase orientation, according to Prevorsek’s three phases model [28,29,30]. The tensile stress arises from the number and orientation of amorphous extended chains connecting the microfibrils, called “interfibrillar taut tie molecules”. Microfibrils are composed of crystalline blocks embedded into slack amorphous chain domains and intrafibrillar extended chains. The higher the orientation and the amount of the taut ties molecules, the higher the tensile stress. Therefore, molecular orientation is of primary importance to the mechanical properties of melt-spun filaments. In this study, the relationship between the final properties and the filament structure is consistent with the literature. The structure characterization through crystallinity, crystalline orientation, and mesophase characterization is globally consistent with the mechanical properties: a lack of a crystalline and mesophase fraction and orientation is observed for low mechanical properties samples compared to the reference. On the contrary, the reference has the highest crystallinity, mesophase fraction, crystalline orientation, and mesophase orientation, resulting in the highest mechanical properties.

In addition, it can be observed that opaque PET addition impacts the mechanical properties more negatively than ${\mathrm{TiO}}_{2}$ addition at equivalent content of the filler. It is due to a lack of molecular orientation, suggested by the lower crystallinity and crystalline orientation of opaque PET systems. However, the mesophase quantification and orientation measurements do not confirm this hypothesis. Indeed, according to mechanical results, opaque PET addition should reduce mesophase fraction more than ${\mathrm{TiO}}_{2}$ addition in standard rPET at the same amount of filler. Figure 7c shows that they behave similarly. It can be suggested that this measurement is not precise enough to show the differences between the two sets of systems. As mentioned earlier, this analysis is difficult to perform accurately on crystallized samples [28]. The crystalline contribution, which needs to be subtracted by peak deconvolution for this analysis, may have been underestimated for opaque samples or overestimated for standard rPET + ${\mathrm{TiO}}_{2}$ samples. In this work, the degree of crystallinity and crystalline orientation was more precisely assessed than the mesophase fraction and is more consistent with the mechanical properties results. As all these characteristics come from the same phenomena, namely molecular orientation during spinning, they are preferred to explain mechanical properties.

Nonetheless, the lack of molecular orientation during melt spinning is mainly due to ${\mathrm{TiO}}_{2}$ particles, as observed by the results of the systems composed of the reference filled with ${\mathrm{TiO}}_{2}$ from the masterbatch. It can be suggested that ${\mathrm{TiO}}_{2}$ submicron particles physically disturb the chain orientation during stretching, as described by Taniguchi et al. [10]. However, opaque PET formulation shows clearly lower mechanical properties than REF +${\;\mathrm{TiO}}_{2}$ from the masterbatch, meaning that the amount of filler is not the only characteristic impacting the spinning process. The opaque PET matrix’s rheological properties or chemical composition may also play an important role. Since opaque rPET is a blend of multiple commercial formulations, co-monomers, contaminants, or additives’ compositions remain unknown and may have an impact on spinnability.

Even though the molar mass of each formulation is very similar, along with the viscosity, the raw materials’ rheological properties are slightly different in elongation. The opaque PET grade melt strength is lower than the reference. This reduced elongational viscosity could be responsible for the poor orientation during spinning, as the amount of stress undergone by the filament during spinning determines the degree of crystallinity and the molecular orientation [27,40]. Then, this decrease in molecular orientation results in a lower tenacity. In addition, the lower draw ratio at break suggests a lower drawability and, consequently, the impossibility of increasing the take-up speed, i.e., the stress in the spinline.

Another point impacting the molecular orientation, and thus the tenacity is the differences in the ratio of elastic versus viscous behavior observed in shear rheology at low deformation (see Figure 3b). Opaque PET has a lower tan δ than standard rPET with and without TiO_2_ addition. It has been previously observed that a higher elastic behavior decreases the spinnability of the polymer melt, although it was for much higher behavior differences [39]. In addition, a lower tan δ can suggest that the molar mass distribution may be higher for opaque PET formulation than REF + ${\mathrm{TiO}}_{2}$ formulation for the same average molar mass. A wider molar mass distribution centered on the same average value induces a shorter chain length, which can hinder molecular orientation during spinning by the relaxation phenomenon and Brownian motion, according to Ziabicki et al. [4].

## 4. Conclusions

The pilot high-speed spinning tests carried out in this work show that opaque rPET is less spinnable than standard rPET, supporting the a priori hypothesis developed with lab shear and extensional rheology measurements. Indeed, it was observed that:

- The degree of crystallinity, along with the crystalline orientation, is lower for opaque PET compared to standard rPET, from 40% to 30% and 0.83 to 0.61, respectively.
- Mesophase fraction seems to decrease from 25% to 15% due to a reduced molecular orientation during high-speed spinning.
- The tenacity of the melt-spun filaments decreases with the addition of opaque PET, from 29 cN/tex without opaque PET to 19 cN/tex at 100% opaque PET. The formulation containing TiO_2_ from the commercial masterbatch and no opaque PET follows the same results but to a lesser extent, with a tenacity of 23 cN/tex at 6.8% of filler (6% of TiO_2_).
- The more TiO_2_ is added, the lower the crystallinity, molecular orientation, and tenacity.
- Opaque PET filaments have lower properties than standard rPET filled with TiO_2_ from the masterbatch at the same filler content.

These results show that (i) TiO_2_ particles are partially responsible for the structure modification initiated by opaque PET leading to poor filaments properties, and (ii) the particle amount is not the only characteristic modifying spinnability. Indeed, opaque PET has a more negative impact on spinning than TiO_2_ from the masterbatch blended into standard rPET at the same filler content.

It was observed that the opaque PET matrix has a higher elastic behavior and a lower elongational viscosity than the standard matrix. Both results are often depicted as negative for the spinning process, which could explain the results of this work. Indeed, the lower elongational viscosity of opaque PET may induce lower stress in the spinline resulting in lower molecular orientation. The higher elastic behavior can induce more die swell and less chain entanglement after the die, decreasing the effectiveness of drawing on molecular orientation. However, the systems studied are blends of multiple commercial formulations, so the co-monomers, contaminants, or additives’ precise composition remains unknown and may also have impacted the spinnability.

As a perspective, as both particles and matrix impact the opaque rPET spinnability, its improvement could arise both from particle-matrix interaction modifications or matrix molar mass increase. However, the filler volume fraction is low, so the variations of particle-matrix interactions may be complex to study. On the other hand, the molar mass increase can lead to a reduction in the process window.

## Figures and Tables

**Table 1:** Properties of the raw materials: Standard rPET and opaque rPET.

Name	Standard rPET	Opaque rPET
composition	rPET transparent flakes	Opaque rPET flakes
Filler content (wt %)	0.8	5.7
Intrinsic viscosity (IV) ^1^ (dL/g)	0.82	0.73
$\eta_{0}\;$^1^ (Pa.s) at 280 °C	175	175
Melting temperature (°C)	246	252
Crystallization temperature (°C) at 10 °C/min	192	202

^1^ The calculation of IV and $\eta_{0}$ is detailed later in this paper.

**Table 2:** Characteristics of the extruded blends between standard and opaque rPET matrices.

Name	REF	30%-O	50%-O	100%-O
Composition	standard rPET	standard rPET + 30% opaque PET mix	standard rPET + 50% opaque PET mix	100% opaque PET mix
filler content (wt %)	0.8	2.5	3.3	5.7
$\eta_{0}\;$(Pa.s) at 270 °C	56	67	86	81
Intrinsic viscosity (IV) (dL/g)	0.56	0.56	0.55	0.55

**Table 3:** Characteristics of the extruded blends between standard rPET and masterbatch PET/TiO_2_ 50/50.

Name	REF	1%-Ti	3%-Ti	6%-Ti
composition	standard rPET	$\mathrm{standard}\;\mathrm{rPET}+1\%\;{\mathrm{TiO}}_{2}$	$\mathrm{standard}\;\mathrm{rPET}+3\%\;{\mathrm{TiO}}_{2}$	$\mathrm{standard}\;\mathrm{rPET}+6\%\;{\mathrm{TiO}}_{2}$
$\mathrm{amount}\;(\mathrm{wt}\;\%)\;\mathrm{of}\;\mathrm{PET}/{\mathrm{TiO}}_{2}$ 50/50 masterbatch	0	2	6	12
filler content wt %	0.8	1.8	3.8	6.8
$\eta_{0}\;$(Pa.s) at 270 °C	56	65	57	77
Intrinsic viscosity (IV) (dL/g)	0.56	0.56	0.53	0.52

**Table 4:** Main properties of the two sets of melt-spun filaments and the reference.

Name	REF	1%-Ti0_2_	3%-Ti0_2_	6%-Ti0_2_	30%-O	50%-O	100%-O
composition	Standard rPET	$\mathrm{Std}-\mathrm{rPET}+1\%\;{\mathrm{TiO}}_{2}$	$\mathrm{Std}-\mathrm{rPET}+3\%\;{\mathrm{TiO}}_{2}$	$\mathrm{Std}-\mathrm{rPET}+6\%\;{\mathrm{TiO}}_{2}$	Std-rPET + 30% opaque rPET	Std-rPET + 50% opaque rPET	100% opaque rPET
Filler content (wt %)	0.8 ± 0.2	1.8 ± 0.2	3.8 ± 0.3	6.8 ± 0.5	2.5 ± 0.2	3.3 ± 0.3	5.7 ± 0.3
Degree of crystallinity (%)	40.3 ± 1	40.0 ± 1	37.1 ± 1	35.4 ± 1	39 ± 1	36.3 ± 1	29.9 ± 2
Orientation factor	0.83	0.80	0.76	0.74	0.82	0.80	0.61
Mesophase fraction (%)	25	23	18	11	21	21	14
Tenacity (cN/tex)	29 ± 1	28 ± 2	24.5 ± 2	23 ± 1	26.5 ± 1	23 ± 1	19 ± 1
Deformation at break (%)	58 ± 5	55 ± 10	59 ± 6	68 ± 4	67 ± 7	73 ± 5	89 ± 8

## Data Availability

The data is available on request from the corresponding author.
